# Pain management in cervical cancer

**DOI:** 10.3389/fonc.2024.1371779

**Published:** 2024-04-25

**Authors:** Sebastián Aguiar-Rosas, Ricardo Plancarte-Sanchez, B. Carolina Hernandez-Porras, Jorge García-Andreu, Brenda Olivia Lezcano-Velazquez, Ignacio Reyes-Torres, Silvia Alarcón-Barrios

**Affiliations:** ^1^ Pain Clinic, Instituto Nacional de Cancerología, Mexico City, Mexico; ^2^ Independent Researcher, Mexico City, Mexico; ^3^ Star Medica Hospital, Queretaro, Mexico; ^4^ Independent Researcher, Queretaro, Mexico; ^5^ Independent Researcher, Saltillo, Mexico

**Keywords:** cervix cancer, uterine cervix cancer, pain, cancer pain, treatment, opioids, interventional pain

## Abstract

Cervical cancer (CC) occupies the second place in incidence and mortality among women in México. Despite this, Cervical Cancer continues to have a late diagnosis which leads to a high rate of complications. Pain represents the most feared and disabling symptom, being present in up to 86% of patients with advanced disease. The approach to managing pain in this population has not been studied and described to a full extent. In addition, there is a pressing need to provide concise recommendations to promote adequate pain control. We performed a review of the literature in CC and had experts in the field of pain management evaluate the evidence found. We then issued relevant recommendations on pharmacology and interventional pain management. Thus, the approach to pain management must be comprehensive and individualized, considering the timely and appropriate use of pharmacologic treatment as well as interventional procedures.

## 1 Introduction

CC is potentially treatable, if detected during the early stages of the disease; however, despite advances in screening and treatment, it remains a leading cause of cancer death among women worldwide, as reported by GLOBOCAN 2020. In Mexico, it is the second leading cause of incidence and mortality in women (1). Diagnosis of cancer in advanced stages often results in severe complications and debilitating symptoms for the affected individual. Among these symptoms, pain is predominant, affecting approximately 86% of patients with advanced-stage cancer, 59% with locally advanced cancer, and up to 33% of cancer survivors (2, 3). The adequate management of pain in these patients continues to be a challenge potentially leading to emotional and physical distress, as well as a lack in quality of life (4–6). The International Association for the Study of Pain (IASP) definition of pain from 2020 provides valuable guidance in identifying pain (7). It is defined as an unpleasant sensory and emotional experience associated with, or resembling that associated with, actual or potential tissue damage. Also taking into account the following points:

- Pain is always a personal experience influenced by biological, psychological, and social factors.
- Pain and nociception are different phenomena. Pain cannot be inferred from activity in sensory neurons.
- Through their life experiences, individuals learn the concept of pain.
- A person’s report of an experience as pain should be respected.
- Although pain usually serves an adaptive role, it may have adverse effects on function and social and psychological well-being.
- Verbal description is only one of several behaviors to express pain; inability to communicate does not negate the possibility that a human or a nonhuman animal experiences pain.

Each patient’s experience of pain is unique, mandating a comprehensive evaluation. The intensity and characteristics of pain can be evaluated using several scales and questionnaires (8). These instruments assist in categorizing the type of pain, including neuropathic, nociceptive (somatic and visceral) as observed in CC patients. Commonly employed scales include the visual analog scale (VAS), numeric rating scale (NRS), and verbal rating scale (VRS). Questionnaires, like the McGill Pain Questionnaire and the Douleur Neuropathique-4 (DN4), can help identify neuropathic pain (9).

CC patients may suffer from nociceptive pain, neuropathic pain and mixed pain. Pain may originate from tumor invasion to viscera in the pelvic cavity, as well as infiltration to nearby tissue, nervous plexuses, peripheral nerves and even lead to bone metastasis in some cases. It is not uncommon for patients with CC to undergo certain procedures during their treatment like placement of percutaneous nephrostomies in order to improve renal function, unfortunately these procedures are associated with discomfort and pain. Lastly treatments used in CC may also be an important source of pain (10).

Radiation therapy (RT) has become a fundamental part in CC treatment and has increased survival rates among this group of patients. It may however be a potential cause of acute and in some instances, chronic pain. Damage of deoxyribonucleic acid (DNA) is one of the mechanisms of action of RT causing irreversible damage to cancer cells, it also induces an increase of several pro-inflammatory cytokines (TNF-alpha, IL-6, TGF-B and others), thus causing damage to nearby healthy tissue as well. Tissue with rapid cellular turnover as is the case with the epidermis and the gastrointestinal mucosa can be affected in an acute manner, presenting with dermatitis or acute enteritis. Other tissue with slow turnover like nerves may present with late adverse effects. In the case of CC, patients primarily present pain due to enteritis, dermatitis and neuropathic pain which may include radiation-induced plexitis (11, 12).. Chemotherapy plays a key role in CC, treatment, unfortunately these drugs may lead to microtubule disruption, mitochondrial damage, altered ion channel activity, neuroinflammation and myelin sheath destruction. Up to 68.1%, 60%, and 30% in the first, third, and sixth months after chemotherapy, respectively may present chemotherapy-induced peripheral neuropathy (CIPN). Platinum-based drugs (70–100%) and taxanes (up to 87%) pose the highest risk in CIPN development and drugs like paclitaxel can cause an acute neuropathy within days after each dose (13, 14).

Despite the available resources for pain management in cancer patients, there is a lack of specific guidance for Cervical and Uterine cancer (CUC) patients. This review aims to develop evidence-based recommendations to provide clinicians insight on the available treatments to effectively manage pain in CC patients, improving their quality of life and overall well-being.

## 2 Methods

A comprehensive literature review investigated existing approaches for managing pain in CC patients. The search terms “Cervix Cancer”, “Uterine Cervix Cancer”, “Pain”, “Cervix Cancer”, “Cancer Pain”, “Treatment”, “Opioids”, and “Interventional Pain” were utilized in PubMed. The goal was to focus on pelvic cancer pain, particularly in cervical cancer patients, as this area is often overlooked, the search included articles published in the year 2000 onward. After an initial screening of titles and abstracts, two authors pre-selected articles that met inclusion criteria. Subsequently, experts in the field of pain management evaluated the information using the Grading of Recommendations, Assessment, Development, and Evaluations (GRADE) to identify the most relevant and current literature. Studies such as clinical cases, case series, letters to the editor, blocking technique descriptions, animal experiments, and those deemed weak recommendations or insufficient evidence were excluded. Out of 1251 articles found in PubMed, 25 were chosen for in-depth review, with 11 subsequently deemed most relevant by an expert panel. These articles are summarized in **Table 1**. Based on this review, specific recommendations were developed for pharmacological and interventional treatments and potential complications associated with the therapy.

**Table 1:** Evaluation of the quality of studies on pain in cervical cancer.

Author and year	Study type	Aim	Number of patients	Follow-up	Results	Quality of evidence	Expert recommendation
Wang J (15), 2020	RCT	Effect of S-ketamine on pain/depression management in patients with CC undergoing total laparoscopic hysterectomy.	Total 417 G I Control (n=105) 50 ml of SS G II: Racemic ketamine (n=104)0.5mg/kg/50ml G III: high-dose ketamine (n=104) 0.5mg/kg/50ml S-Ketamina G IV: Low doses of Ketamine (n=104) 0.25mg/ke/50ml	Assessment at 1,2,3,5 and 7 days after total hysterectomy.	Significant decrease in VAS in all treatment groups compared to the control group (p< 0.05) No difference between the high-dose S-ketamine, low-dose S-ketamine, or racemic ketamine group.	High	Weak, in favor
Meng F (16), 2018	Observational retrospective, retrolective study	Effectiveness of acupuncture in pain due to CC	Total 64 G I:32 patients with acupuncture G II:32 control patients	Assessment prior to treatment and after 14 days	Significant decrease in NRS 2.3 in the intervention group vs 4.5 in the control group (p=< 0.01)	Low	Weak, in favor
Krakauer E (17), 2021	Retrospective, descriptive.	Recommendations by a group of experts to reduce refractory symptoms in patients with CC				Low	Weak, in favor
Blackburn L (18), 2021	RCT	Study in patients receiving brachytherapy, comparing usual care vs. the same care + aromatherapy and reflexology.	Total 41 G I:22 patients intervention group with reflexology and aromatherapy G II:19 control patients, usual care during brachytherapy	Assessment in 5 stages upon arrival at the clinic, after insertion of the brachytherapy equipment, pre-reflexology, post-reflexology, after removal of the equipment.	Patients in the intervention group showed a significant decrease in pain after receiving reflexology compared to the control group (p < 0.0001).	Moderate	Weak, in favor
Erdine S (19), 2003	Non RCT	Effectiveness of a new approach for superior hypogastric plexus block.	Total 20 patients with pelvic pain of oncological origin	Assessment prior to the procedure, 24 hours after the procedure, every month for 3 months.	12 patients with a significant decrease in pain at 24 hours and at one month (p<0.05) during months 2 and 3 without significant changes.	Moderate	Strong, in favor
Gamal G (20), 2006	RCT	Effectiveness of a new vs. old approach to perform superior hypogastric plexus block.	Total 30 G I:15 patients underwent transdiscal superior hypogastric plexus lysis G II:15 patients undergoing lysis of the superior hypogastric plexus by the classical approach	Assessment prior to the procedure, 24 hours after the procedure, every month for 3 months.	Significant reduction in intervention time of transdiscal technique vs classic technique (p< 0.05) No significant differences in the decrease in VAS or daily morphine consumption.	High	Strong, in favor
Plancarte R (21), 1990	Cohort	Effectiveness of superior hypogastric plexus neurolysis in patients with pelvic cancer	28 patients with pelvic pain of oncological origin	Assessment prior to the procedure, at 30 minutes, 1, 2, 4, 8 and 24 hours after the procedure, every month until the patient died.	70% reduction in baseline pain.	Low	Strong, in favor
Rocha A (16), 2020	Retrospective cohort	Effectiveness of superior hypogastric plexus neurolysis in patients with pelvic cancer	180 patients with abdominal and pelvic pain of oncological origin	Retrospective follow-up to 3 years after the procedure.	59.4% patients with a decrease in pain of at least 50% at one month, 55.5% at 3 months and 48.8% at 6 months.	Low	Strong, in favor
Amr Y (22), 2014	Multicenter RCT	Effectiveness of early sympathectomy to reduce pain, reduce opioid consumption and improve quality of life in cancer patients	**Total:** 109 patients **G I:**54 patients with neurolysis as step 2 of the WHO ladder (early neurolysis) **II:**55 patients with neurolysis as step 4 of the WHO ladder (late neurolysis)	Follow-up until death of patients undergoing the procedure.	Significant decrease in pain in group I compared to group II up to 6 months. (p = < 0.0001) Decrease in opioid doses significantly in group I compared to group II. (p= < 0.0001)	High	Strong, in favor
Mishra S (23), 2013	RCT	Effectiveness of Ultrasound-Guided Superior Hypogastric Plexus Neurolysis in Patients With Cancer Pain	Total: 50 patients G I:25 patients with oral morphine G II:25 patients with ultrasound-guided superior hypogastric plexus neurolysis	First month: Weekly Second month: Every two weeks Third month: monthly follow up	Significant decrease in VAS at week, 1 week, 1 month, 2 months and 3 months. Non-significant decrease in VAS between group I and II at 3 months.	High	Weak, in favor

Niveles de Evidencia: A, Alta; B, Moderada; C, Baja; D, Muy Baja.

## 3 Results

### 3.1 Pain treatment in CC

In 1986, the World Health Organization (WHO) introduced the analgesic ladder to guide and standardize pain treatment for cancer patients. The first three steps of this ladder suggest suitable analgesics based on the pain intensity reported by the patient. Over the years the ladder has gone through changes to include minimally invasive procedures, such as epidural or intrathecal analgesia, peripheral nerve blocks, neuromodulation, ablative blocks, and other surgical techniques, which are commonly employed for managing pain, including pelvic pain in patients with CC (24–26). Despite these modifications, the WHO analgesic ladder still has fundamental gaps regarding the treatment of pain in cancer patients. For instance, it does not address the etiology of pain that can be present and focuses primarily on the intensity of pain to guide treatment.

#### 3.1.1 Pharmacological treatment

Cancer pain management is specific to its type, intensity, and characteristics. Various classes of drugs are available for pain relief, including non-steroidal anti-inflammatory drugs (NSAIDs) (27, 28), acetaminophen, opioids, and adjuvants. NSAIDs and acetaminophen are used for mild pain, in the case of CC these medications are usually indicated in the early stages of the disease and when acute pain presents, for instance undergoing procedures like percutaneous nephrostomies or catheter placement. NSAIDS can also be administered in patients that present with musculoskeletal pain secondary to tumor invasion or in cases of acute dermatitis secondary to radiotherapy. A significant number of patients with CC may present with deterioration in renal function and NSAIDs should be used with caution, avoiding their chronic use. Opioids are typically used for moderate to severe pain, and their dosage is titrated as needed. Weak and strong opioids are indicated in the context of visceral pain, somatic pain (musculoskeletal involvement), neuropathic pain and mixed pain secondary to tumor invasion. Acute or chronic pain secondary to adverse events due to chemotherapy or radiotherapy like CIPN, enteritis, dermatitis and radiation-induced plexitis may also be treated with opioids (29–31). Adjuvants are medications that are not typically used for pain relief but have characteristics that help in pain management (32). They include tricyclic antidepressants, serotonin reuptake inhibitors, anticonvulsants, local anesthetics, NMDA receptor antagonists, topical therapies, corticosteroids, bisphosphonates, and cannabinoids. Adjuvants are generally used to treat neuropathic pain that appears after radiotherapy, chemotherapy and in cases of tumor infiltration as well (28, 33–37). (See **Table 2**)

**Table 2:** Pain treatment approach in cervical cancer.

Type of Pain	Etiology	Characteristics	Pharmacotherapy	Interventional Management	Non-pharmacological treatment
Visceral Pain (38) (nociceptive)	- Tumor compression -Radiotherapy Non-cancer pain (functional motility disorders)	Secondary to injury or organ dysfunction. It is deep, continuous, poorly located and radiates even to areas far from the point of origin. It is usually accompanied by vegetative symptoms (nausea, vomiting, sweating).	Paracetamol (29) -Limit use to 3 grams per day. -Offers limited pain relief. NSAIDs (29) -Non-selective COX-2 inhibitors -Selective COX-2 inhibitors Opioids (29) Mild pain: -Consider the use of weak opioids. - Titrate individually. Moderate to severe pain: -Consider the use of major opioids. - Titrate individually. Consider the use of buprenorphine or fentanyl in cases of renal impairment (29–31) Adjuvants *Antidepressants (24) tricyclic *Serotonin and norepinephrine reuptake inhibitors (29).^ ^ *Anticonvulsants (29) * Intravenous lidocaine (35) *Intravenous ketamine (36) *Corticosteroids (37) *Octreotide (39) *Anticholinergics (39)	Neurolysis of the superior hypogastric plexus (cancer pelvic pain) (16, 21, 25, 26) Impar ganglion block (perineal cancer pain) (40, 41) Sacral neurolysis using radiofrequency or intrathecal neurolytics (perineal cancer pain) (42) Sacral neurostimulation (perineal cancer pain) (43) Implantable therapy (44)	Psychological therapy (38) Psychosocial support Integrative therapies: -Consider aromatherapy during treatment with brachytherapy, it could reduce anxiety and pain (45). -Music therapy (18) -Healing touch therapy (46) -Consider reflexology during treatment with brachytherapy, it could reduce anxiety and pain (18). -Acupuncture: It could improve pain in patients with CC with a low incidence of side effects (17). -Mindfulness: Could improve pain via non-opioidergic modulatory pathways (46–49)
Somatic Pain (38) (nociceptive)	-Tumor compression -Treatment: *Placement of catheters or nephrostomies. -Diagnosis: *Biopsy taken. -Pain not related to cancer (osteoarthritis or degenerative diseases)	Caused by the activation of nociceptors in response to a stimulus (injury, inflammation, infection, disease). It is characterized by being well located and although it is frequently sharp, its typology varies from one patient to another.	Paracetamol (29) -Limit use to 3 grams per day. -Offers limited effectiveness. NSAID (29) Opioids (29) Soft pain: -Consider the use of weak opioids. - Holder individually. Moderate to severe pain: -Consider the use of major opioids. - Holder individually. - Consider the use of treatment with buprenorphine or fentanyl in cases of renal impairment (29–31) Adjuvants * Tricyclic Antidepressants (29) *Serotonin and norepinephrine reuptake inhibitors (29)^ ^ *Anticonvulsants (29) *Intravenous lidocaine (35) *Intravenous Ketamine (36) *Corticosteroids (37) *Muscle relaxants (50)	Neurostimulation (Spinal Cord, DRG or peripheral devices) (44, 51)	
Neuropathic pain (38, 52)	-Tumor compression -Treatment: Radiotherapy Chemotherapy	Direct central or peripheral injury by the tumor. Secondary to chemotherapy: pain associated with numbness and tingling. Generally presenting with a sock or glove distribution.	First line (33): -Gabapentin 1200-3600mg/day -Pregabalin 300mg-600mg/day -Duloxetine 60-120mg/day -Venlafaxine 150-225mg/day. Second line (33, 34): • Capsaicin patches: one to four patches on the painful region once a day for 12 hours • Lidocaine patches: one to three patches on the painful region once a day for 12 hours • Tramadol 200-400mg/day • Major opioids (34), individual titration.	Neurostimulation (Spinal Cord, DRG or peripheral devices) (44, 51)	

NSAID, Non-steroidal anti-inflammatory drugs.

TD, Transdermal.

The treatment of cancer pain should consider not only the intensity of pain but also its type (nociceptive, neuropathic, or mixed) (38, 39). The medication and dosage should be tailored to the patient’s characteristics, cases of mixed pain generally require a combination of the available options described (24, 29). Patients with special conditions such as impaired kidney or liver function or constipation may need special considerations, such as the use of buprenorphine or fentanyl (30, 31, 53, 54). (See **Table 2**)

#### 3.1.2 Interventional treatment

An important number of patients may experience adverse effects or inadequate response to the pharmacologic treatment available (32). Therefore, implementing interventional pain management strategies can be beneficial, aiming to enhance pain control and potentially reduce opioid consumption (16). Among patients with cancer pain, particularly those with visceral pain invading the pelvis, such as in CC, commonly used intervention procedures include the superior hypogastric plexus and the impar ganglion block. Both procedures were described in 1990 for the first time by Plancarte et al. They continue to be a useful intervention in early and advanced stages of CC for managing pelvic cancer pain, reducing opioid consumption and their possible side effects. In advanced stages of CC with retroperitoneal invasion, the trans-discal and transvascular approaches for performing superior hypogastric plexus neurolysis (SHPN) achieve similar effects (21, 25, 26, 45).

In many cases of CC, patients present with radiation of pain to the genital or perineal region. The impar ganglion is a single retroperitoneal structure near the sacrococcygeal junction, contributing to pelvic, genital, and perianal innervation. Consequently, since the initial description of the impar ganglion neurolysis in 1990, various techniques and substances have been used, aiming to alleviate pain in this anatomical region. This procedure has demonstrated pain relief of up to 70% in multiple studies (40). These interventions should be considered in most cases of CC, particularly in the early stages presenting with perineal pain. Appropriate execution of these procedures is essential for successful pain management (4, 40, 41).

Finally, it is crucial to execute a thorough evaluation of the patient before performing any of the procedures mentioned. A detailed physical exam directed toward determining the etiology and type of pain presented as well as a previous detailed inspection of imaging studies to determine the best approach in each particular case depending on tumor location and anatomical variations. Some of the absolute contraindications include patient refusal, local or systemic infections, allergies to any of the medications used during the procedure including contrast media, patients with coagulopathy (Platelet count < 50,000 and INR > 1.5), lack of technical experience performing any procedures mentioned and uncertainty regarding the patient´s diagnosis. It is also important to consider patients with neutropenia due to chemotherapy and potential risk of infection, patients that cannot tolerate the adequate position necessary to perform the procedure, bowel obstruction and to avoid puncture of the tumor. Patients under treatment with anticoagulants should also have medication suspended and restarted in a timely manner in order to avoid risk of bleeding. In general, the benefits of interventional procedures should always outweigh potential risks (55, 56). (See **Table 2**)

#### 3.1.3 Integrative therapies

Anxiety, discomfort and pain are significant issues for patients with CC due to tumor activity and treatment undergone by these patients. Various non-pharmacological therapies have shown promise in alleviating symptoms associated with the disease or its treatment. Acupuncture, a minimally invasive technique, has effectively reduced pain in CC patients (17). Psychological therapy, reflexology, and aromatherapy have also been reported to provide relief from anxiety and pain, particularly before brachytherapy treatment (18, 57).

Other non-pharmacological approaches, such as meditation, music therapy, and tai chi, have also exhibited benefits in reducing pain, anxiety, depression, and stress in patients. These therapies, when combined with conventional pain management strategies, can offer a comprehensive approach with minimal side effects, making them suitable for consideration in the treatment of CC patients (17, 18, 57) (See **Table 2**).

## 4 Discussion

Pain in patients with CC is a prevalent and debilitating symptom affecting a large proportion of patients. Research has consistently highlighted the prevalence of cancer pain in cervical cancer patients. In 2007, a pain prevalence of 64% in advanced disease and 59% in locally advanced disease was reported. Subsequent studies in 2016 yielded similar results, 66.4% and 55%, respectively (3, 58). Current data (2, 59, 60) also indicates a wide range of pain prevalence, from 53.8% to 68%, across diverse populations, including the Mexican population. Approximately 96% of patients experience mild pain, while 84% report moderate to severe pain during the disease course (7).

Pain management in CC patients poses challenges for healthcare professionals. It requires individualized treatment plans encompassing pharmacological and non-pharmacological interventions (29, 33, 38, 50). The choice of treatment depends on the distinct types of pain experienced by the patient, the availability of medications, the patient’s resources, and the regional healthcare context. As mentioned, NSAIDs and Acetaminophen are usually used in cases of mild nociceptive pain that may be secondary to tumor activity or as an adverse event as is the case of acute dermatitis after radiotherapy or after catheter or percutaneous nephrostomy placement. Opioids, due to their effectiveness, ease of titration, and favorable risk-benefit profile, constitute the mainstay of pharmacological pain management in patients with moderate to severe cancer pain secondary to tumor invasion of viscera in the pelvic cavity, the musculoskeletal system and nerves that may be compromised. In the case of neuropathic pain secondary to tumor invasion, chemotherapy or radiotherapy opioids are the second or in some cases the third line of choice (15, 52, 61–63). There is a lack of non-inferiority studies comparing the efficacy of different opioids in CC. The selection of an appropriate opioid should consider various barriers that influence access and availability, including regulatory and legal restrictions, knowledge gaps, and economic constraints as well as comorbidities such as renal failure that may limit the use of some of these analgesics (29, 64, 65).

Adjuvant therapies like antidepressants, gabapentinoids, lidocaine or ketamine infusions are often employed to address neuropathic and mixed pain in locally advanced and advanced cervical cancer, as mentioned before these medications are widely used not only in neuropathic pain secondary to tumor infiltration but also to treat possible painful adverse events that arise from chemotherapy and radiotherapy.

Pain management in CC is frequently complicated and occasionally challenging to control using pharmacological treatments. Consequently, interventional therapies, such as SHPN, are used. SHPN was first described as a traditional technique by Plancarte et al. in 1990, along with two alternative approaches (trans-discal and transvascular) (21, 26). Erdine et al. in 2003 describes in depth the trans-discal approach; offering a significantly faster procedure with similar results as the classic approach (19). WHO suggests SHPN and other interventional procedures as a final resort in pain management, a study by Amr Y et al. in 2014 showed significant pain relief and reduced opioid consumption when performed in patients with pelvic cancer pain during early stages of disease (22). In 2015 a systematic review by Mercadante et al. suggested a low recommendation level for SHPN due to the scarcity of high-quality studies. Recent research (Rocha et al., 2020 and Pérez-Moreno et al., 2020) however, indicates that SHPN is an effective technique for managing oncological pelvic pain (4, 16, 66).

Technological advances have led to novel pain management techniques, such as ultrasonography in SHPN, proving safer for cancer pain patients (23, 67). Similar pain relief and a lower rate of severe side effects have been observed using ablative radiofrequency compared to chemical neurolysis for sacral nerve pain (42). Moreover, pulsed radiofrequency combined with SHPN at the sacral level enhanced pain relief without increased adverse effects (43). Additionally, intrathecal therapies and neurostimulation devices have shown promising results in cancer pain management (44, 51). However, more controlled studies in patients with CC are needed to explore the full efficacy of these treatments, and this is an area of opportunity for future research.

Non-pharmacological therapies, including psychosocial support and integrative therapies (mindfulness, tai chi, acupuncture among others), offer additional pain relief in patients with CC when used alongside pharmacological and interventional measures. These therapies have effectively provided relief with minimal side effects, making them suitable as complementary support therapies for this patient population (17, 18, 32, 38, 46). In the case of mindfulness, studies using magnetic resonance imaging have allowed the identification of different areas of the brain involved in the perception and modulation of pain after such therapies. These areas identified include the orbitofrontal cortex, the anterior cingulate cortex and the anterior insular cortex. Mindfulness meditation appears to not only target pain via multiple unique non-opioidergic modulatory pathways but to also mitigate the psychological risks of developing opioid use disease, therefore this could prove useful in providing a more adequate pain control in CC patients and in avoiding opioid misuse in this population (46–49).

### 4.1 Recommendations

Pharmacologic treatment:

1) Use acetaminophen to start treatment in cases of mild pain. 1C
2) Use of NSAIDs to start treatment in mild to moderate pain or inflammatory process. 2C
3) Use of acetaminophen and weak opioids in mild to moderate pain. 2C
4) Use morphine as the first option in cases of moderate to severe pain. Consider the use of another significant opioid if morphine is not available. 1A
5) Opioid titration must be carried out on an individualized basis. 1A
6) In case of breakthrough pain, use transmucosal Fentanyl. Use of fentanyl is also recommended in cases of opioid tolerance. 2A
7) Make use of neuromodulators (gabapentinoids, tricyclic antidepressants, serotonin and norepinephrine reuptake inhibitors, local anesthetics, and ketamine) in cases of neuropathic or mixed pain. 1A
8) Indicate prophylactic treatment for nausea and timely treatment in case of other events—adverse events associated with opioids. 1A
Interventional therapy:
9) Employ early interventional approaches to control pain: Neurolysis of the superior hypogastric plexus and impar ganglion. 1A
10) Use of implantable therapy and neurostimulators. 2B
Non-pharmacologic therapy:
11) Integrative therapies (aromatherapy, reflexology, acupuncture, mindfulness) can play a role as adjuvants for pain control. 2C

## 5 Conclusion

Effective pain management in cervical cancer patients necessitates a comprehensive and individualized approach. A thorough assessment of the pain characteristics is paramount in selecting targeted treatments. Timely management of potential side effects is crucial for optimal outcomes.

## Author contributions

SA-R: Investigation, Writing – original draft, Writing – review & editing. RP-S: Supervision, Writing – original draft, Writing – review & editing. BH-P: Writing – original draft, Writing – review & editing. JG-A: Writing – review & editing. BL-V: Writing – review & editing. IR-T: Writing – review & editing. SA-B: Formal analysis, Investigation, Methodology, Writing – original draft, Writing – review & editing.
